# Evaluating the Trends of Bloodstream Infections among Pediatric and Adult Patients at a Teaching Hospital of Kathmandu, Nepal: Role of Drug Resistant Pathogens

**DOI:** 10.1155/2017/8763135

**Published:** 2017-04-06

**Authors:** Narayan Prasad Parajuli, Hridaya Parajuli, Roshan Pandit, Jyotsna Shakya, Puspa Raj Khanal

**Affiliations:** ^1^Department of Clinical Laboratory Services, Manmohan Memorial Medical College and Teaching Hospital, Kathmandu, Nepal; ^2^Department of Laboratory Medicine, Manmohan Memorial Institute of Health Sciences, Kathmandu, Nepal

## Abstract

Bloodstream infections (BSIs) are among the significant causes of morbidity and mortality for patients of all age groups. However, very little is known about the trends of bacterial bloodstream infections and antimicrobial susceptibilities among pediatric and adult population from Nepal. In this study, we have investigated the different etiological agents responsible for bloodstream infections among pediatric and adult patients and the role of drug resistant organisms in these infections at a tertiary care teaching hospital of Kathmandu, Nepal. A total of 3,088 blood culture specimens obtained from pediatric and adult patients suspected to have bloodstream infections were processed by standard microbiological methods. Significant bacterial pathogens were identified by morphological, biochemical, and serological methods as suggested by American Society for Microbiology. In vitro antimicrobial susceptibility testing was performed by Kirby-Bauer disk diffusion method and interpreted according to the guidelines of Clinical and Laboratory Standards Institute. Overall, incidence of bloodstream infections among the suspected patients was 7.48%. Pediatric patients (*n* = 90, 9.37%) were the significant subgroup of patients affected with bloodstream infections compared to adults (*p* < 0.05, CI-95%). Gram positive (*n* = 49, 54.4%) bacteria in pediatric and gram negative bacteria (*n* = 141, 78.7%) in adult patients were the most common isolates for BSI.* Staphylococcus aureus* (*n* = 41, 45.6%) in pediatric patients and* Salmonella enterica* (*n* = 40, 28.3%) in adult patients were the leading pathogens. Trends of antimicrobial resistance among isolated bacterial strains were significantly high in adults compared to pediatric patients. Methicillin resistant* Staphylococcus aureus* (MRSA) (31.4%), extended spectrum beta-lactamase (ESBL) (12.5%), and metallo-beta-lactamase (MBL) (3.9%) producing gram negatives were major resistant strains. Our study shows higher rates of bloodstream infections in pediatric patients compared to adult patients. Alarming rates of antimicrobial resistance among blood culture isolates is a serious issue. Prompt and accurate diagnosis and rational antimicrobial therapy are extremely needed.

## 1. Introduction

Bloodstream infections (BSIs) are defined as the presence of viable infectious microorganism in the bloodstream causing clinical illness [1]. They are among the leading causes of mortality and morbidity worldwide [2]. The term bloodstream infection and bacteremia are synonymously used which generally refer to the significant growth of a microorganism in a blood culture obtained from the patient with clinical signs of infection [3]. In clinical practice, bacteremia may range from self-limiting infections to life threatening septicemia that requires prompt and rational antimicrobial treatment [4]. However, in the developing countries, changing epidemiology, lack of standard antimicrobial guidelines in locality, emergence of antimicrobial resistance, and paucity of good diagnostic facilities are major denominators for surge in BSI associated morbidity and mortality [5].

Alongside, incidence rates of BSI have been found to be bimodal. Increased rates have been observed in extreme ages of life due to poor immune competency as well as the presence of comorbid conditions [6, 7]. Differences in the diagnostic approaches, antimicrobial therapy, and clinical management of BSI among pediatric and adult patients have been described elsewhere [5, 8]. Furthermore, treatment of bloodstream infections in developing world is often empirical, primarily due to the lack of standard therapeutic guidelines and unavailability of susceptibility pattern of the local isolates [3]. Over the years, there has been dramatic shift in the etiology of bloodstream infections with gram negative bacterial dominance. These agents are continuously evolving with novel drug resistant determinants resulting in the poor therapeutic outcomes in BSI [2]. Reports regarding higher prevalence of gram negative isolates causing BSI in South Asian region producing extended spectrum beta-lactamases (ESBL) and carbapenemases are of great concern, as it has major impact on selecting and prescribing the antimicrobial therapy [9–11]. In the relevance of the global studies in the trends of bloodstream infections and increasing antimicrobial resistance, there is a strong need of evaluation of such trends in Nepalese scenario. Therefore, a systematic study was carried out among the pediatric and adult patients to investigate the etiology and trends of bacterial pathogens as well as the role of drug resistant isolates in these infections.

## 2. Materials and Methods

### 2.1. Study Setup

This was a hospital based cross sectional study carried out between March 2015 and August 2016 (over a period of 18 months) at the Department of Microbiology, Manmohan Memorial Teaching Hospital, a tertiary care referral center with 300 patient beds, in Kathmandu, Nepal. During the study, a total of 3,088 patients (pediatric and adults), clinically suspected of bloodstream infections (BSIs), were enrolled. Patients already on antibiotics and repeated samples from the same patients were excluded.

### 2.2. Laboratory Investigations

Patients visiting outpatient departments (pediatric and general medicine) and those admitted in the inpatient units were investigated for bloodstream infections by respective unit physicians. At the onset of fever (>37°C) or in the presence of any clinical symptoms compatible with infection, a blood culture specimen was taken with aseptic technique by cleansing of the collection site with 70% alcohol and subsequently followed by povidone iodine. One mL (for neonates), 5 mL (for children), and 10 mL (for adults) of blood specimen were collected and they were inoculated into brain heart infusion (BHI) broth at the blood to broth ratio of 1 : 10. After incubation, at 37°C for 24, 48, and 72 hours, blind subcultures were made on MacConkey agar and blood agar plates (HiMedia Laboratories, India). The plates were observed for bacterial growth after 24 hrs of aerobic incubation at 37°C. Identification of significant isolates was done by using standard microbiological techniques which involved morphological appearance of the colony, gram's staining reactions, catalase test, coagulase test, and oxidase test with other biochemical and serological properties [12]. Samples were considered sterile, if no growth was observed on subculture after 7 days of aerobic incubation at 37°C. Laboratory confirmed BSI was considered when a bacterial pathogen was recovered from at least one blood culture with promising clinical symptoms.

### 2.3. Antimicrobial Susceptibility Testing

The susceptibility of bacterial isolates against different antibiotics was tested by the disk diffusion method [modified Kirby-Bauer method] on Mueller Hinton agar (HiMedia Laboratories, India) following standard procedures recommended by the Clinical and Laboratory Standards Institute (CLSI), Wayne, USA [13]. Antibiotics that were tested in this study include ampicillin (Amp 10 *μ*g), amikacin (30 *μ*g), gentamycin (Gen 10 *μ*g), ciprofloxacin (CIP 5 *μ*g), levofloxacin (5 *μ*g), trimethoprim-sulfamethoxazole/cotrimoxazole (COT30 *μ*g), cloxacillin (5 *μ*g), cefixime (CFM 5 *μ*g), cefotaxime/ceftriaxone (CTX/CTR 30 *μ*g), ceftazidime (CAZ 30 *μ*g), chloramphenicol (C 30 *μ*g), azithromycin (AZM 15 *μ*g), piperacillin-tazobactam (PIT 100/10 *μ*g), imipenem (IPM 10 *μ*g), meropenem (MRP 10 *μ*g), teicoplanin (30 *μ*g), and polymyxin B (PB 300 U) from HiMedia Laboratories, India. Interpretations of antibiotic susceptibility results were made according to the guidelines of interpretative zone diameters of CLSI [13].* Escherichia coli* ATCC 25922,* Staphylococcus aureus* ATCC 25923, and* Pseudomonas aeruginosa* ATCC 27853 were used as the control organisms for antibiotic sensitivity.

### 2.4. Identification of Multidrug Resistant (MDR) Isolates

Multidrug resistant (MDR) bacterial isolates were identified according to the criteria recommended by joint committee of international experts from European Centre for Disease Prevention and Control (ECDC) and the Centers for Disease Control and Prevention (CDC) [14]. In this study, the isolate resistant to at least one antimicrobial from three different groups of first-line drugs tested was regarded as multidrug resistant (MDR).

### 2.5. Phenotypic Detection of Extended Spectrum *β*-Lactamase (ESBL)

The initial screening test for the ESBL production was performed by using ceftriaxone (CRO 30 *μ*g), ceftazidime (CAZ 30 *μ*g), and cefotaxime (CTX 30 *μ*g) disks (HiMedia, Mumbai, India). If the zone of inhibition (ZOI) was ≤25 mm for CRO, ≤22 mm for CAZ, and/or ≤27 mm for CTX, the isolate was considered a presumptive ESBL producer as recommended by CLSI [13]. Combination disk test (CDT) was used for the phenotypic confirmation of potential ESBL producing strains in which ceftazidime (CAZ) and cefotaxime (CTX) (30 *μ*g each) alone and in combination with clavulanic acid (CA) (10 *μ*g) was used. An increase in zone of inhibition of more than or equal to 5 mm for antimicrobial agent in combination with CA versus its zone when tested alone was considered as ESBL producer [13]. For ESBL standardization,* Escherichia coli* ATCC 25922 and* Klebsiella pneumoniae* ATCC 700603 were used as negative and positive controls.

### 2.6. Phenotypic Test for Metallo-*β*-Lactamase (MBL)

Isolates that were found nonsusceptible to third-generation cephalosporins (ceftazidime), imipenem, or meropenem in Kirby-Bauer disk diffusion method were presumptively considered MBL producers and confirmed by the combined disk method. Briefly, the test inoculums (comparable to 0.5 McFarland standards) were prepared and transferred onto the Mueller Hinton agar plates. In the combination disk test for MBL, two imipenem (IPM) disks (10 *μ*g), one containing 10 *μ*L of 0.1 M (292 *μ*g) anhydrous EDTA (Sigma Chemicals, St. Louis, MO), were placed 25 mm apart. An increase in the zone size of more than or equal to 7 mm for imipenem-EDTA disk compared to imipenem disk alone indicated MBL producer strain as described by Yong et al. [15].

### 2.7. Phenotypic Test for Methicillin Resistant (MRSA) and Inducible Clindamycin Resistant (iMLS_B_)* Staphylococcus aureus*

Methicillin resistant* Staphylococcus aureus* (MRSA) isolates were detected by cefoxitin disk (30 *μ*g) method of CLSI.* S. aureus* isolates were deemed methicillin resistant when the ZOI for cefoxitin was ≤21 mm [13]. Similarly, inducible macrolide-lincosamide-streptogramin-B (iMLS_B_) resistance was detected in* S. aureus* by disk approximation using clindamycin (2 *μ*g) and erythromycin (15 *μ*g) on MHA plates. After overnight incubation, isolates with flattened zone of inhibition adjacent to the erythromycin disk (referred to as a “D” zone) were considered to exhibit inducible clindamycin resistance [13].

### 2.8. Data Analysis

Patient information regarding patient name, age, sex, ward/bed number (if admitted), brief clinical history, duration of hospital stay, history of antibiotic use, and bacterial isolates and their antimicrobial susceptibilities was taken and entered into a computer program. Data analysis was carried out using the Statistical Package for Social Sciences [SPSS™] version 20.0 [IBM, Armonk, NY, USA] and presented in percentage base distribution. Data with *p* value of less than 0.05 (CI-95%) was regarded as significant.

## 3. Results

Overall, a total of 3,088 blood cultures specimens from patients suspected with bloodstream infections were processed. Specimens were from infants (*n* = 386), children (*n* = 574), adults (*n* = 1949), and the elderly (*n* = 179). Male comprised 50.6% of total patients with male to female ratio of 1.02. Out of 3,088 blood cultures during the period, 231 (7.48%) were positive for significant growth of bacterial pathogen suggesting bloodstream infection.

### 3.1. Trends of BSI among Pediatric and Adult Patients

More pediatric patients (9.3%) were found with BSI as compared to the adult patients (6.6%) and this trend was statistically significant (*p *= 0.008, CI-99%). Among all cases, the highest proportion of culture confirmed bloodstream infections were observed among the infants (*n* = 50, 12.9%) followed by the children (*n* = 40, 6.9%), adults (*n* = 131, 6.72%), and elderly (*n* = 10, 5.5%) patients. (Table 1).

In this study, the rate of blood stream infections varied significantly (*p* value = 0.00) between inpatient (22.1%) and outpatient (7.5%) groups. Furthermore, among various inpatient wards, higher blood culture positivity was observed among critical care patients (pediatric ICU: 26.08% and adult: 27.2%) (data not presented).

### 3.2. Trends of Bacterial Isolates

Table 2 illustrates the common bacterial isolates causing bloodstream infections. Gram negative bacteria (65.8%) were the leading pathogenic agents compared to the gram positive bacteria (34.2%) in this study (*p* < 0.05). Also, there was significant difference between the bacterial etiology of BSI among pediatric and adult patients. (Table 1).* Staphylococcus aureus* was the leading pathogen involved in pediatric cases (*n* = 41, 45.6%), while* Salmonella enterica* (*n* = 40, 28.3%) was the common pathogen involved in adult cases of BSI. Furthermore,* Staphylococcus aureus* was isolated among 45 (31%) of 145 blood culture isolates from inpatients which was followed by* Pseudomonas* (*n* = 39) and* Acinetobacter* species (*n* = 30). Among 86 blood culture isolates from outpatients,* Salmonella enterica* was isolated from 46 (53.5%) cases, followed by* Staphylococcus aureus* (*n* = 21) and* Escherichia coli* (*n* = 9).

### 3.3. Trends of Antimicrobial Susceptibilities of Gram Negative Isolates (excluding* Salmonella*)

In this study, susceptibilities of beta-lactam antibiotics, fluoroquinolones, aminoglycosides, and carbapenems towards gram negative isolates from pediatric patients and adult patients were poles apart.* Escherichia coli* isolates from pediatric patients were completely susceptible (100%) to ciprofloxacin, levofloxacin, amikacin, imipenem, and meropenem, but those from adult patients were resistant to ciprofloxacin and levofloxacin (83.3% each), ampicillin (66.7%), cefixime and ceftazidime (66.7% each), gentamycin and amikacin (33.3% each), and imipenem (16.7%), respectively. Similarly, isolates of* Klebsiella* spp. from pediatric patients were highly susceptible (100%) to ceftazidime, amikacin, and carbapenems while those from adult patients were highly resistant (75%) to cephalosporins and fluoroquinolones, 50% resistant to carbapenems, piperacillin-tazobactam, and chloramphenicol, respectively. Similarly, susceptibilities of* Pseudomonas* spp. against ciprofloxacin (25.0% versus 96.7%), levofloxacin (12.5% versus 87%), imipenem (0% versus 22.5%), and chloramphenicol (0% versus 12.9%) also differed considerably among pediatric and adults patients. However, susceptibilities of* Acinetobacter* spp. were also found in the similar trend (Table 3).

### 3.4. Trends of Susceptibilities of* Salmonella enterica* Isolates

*Salmonella enterica* isolates from pediatric patients were highly susceptible to ampicillin whereas 11.7% isolates from adult patients were found resistant to it. In addition, entire isolates were susceptible to cotrimoxazole, cefixime, ceftriaxone, chloramphenicol, and azithromycin in both groups (Table 4). However, more than 70% of the* Salmonella* Typhi and almost all (up to 100%)* Salmonella* Paratyphi A isolates were resistant to fluoroquinolones.

### 3.5. Trends of Susceptibilities of Gram Positive Isolates

*Staphylococcus aureus* revealed high level of resistance among tested antimicrobials (Table 5). Isolates from pediatric patients were highly resistant to ampicillin (80%), erythromycin (51.1%), and clindamycin (51.1%) and less susceptible to cephalexin (37.7%), cloxacillin (37.7%), and imipenem (37.7%), respectively. Similarly, isolates from adult patients were resistant to ampicillin (100%), cotrimoxazole (84.0%), ciprofloxacin (88.0%), erythromycin (84.0%), clindamycin (80.0%), and levofloxacin (68.0%). Teicoplanin, amikacin, and cloxacillin were effective antimicrobials for both groups of patients with staphylococcal BSI. Furthermore, entire isolates (100%) of* Enterococcus* spp. were susceptible to teicoplanin and about 50% susceptible to amikacin, ciprofloxacin, and levofloxacin whereas only 20% and 75% were susceptible to ampicillin (in adult and pediatric patients, resp.).

### 3.6. Trends of Resistance Determinants in Gram Negative Isolates

In this study, high proportions of the gram negative isolate were multidrug resistant (34.8%) (Table 6). About two-thirds (71.4%) of* Klebsiella* spp. and half (50%) of the* Escherichia coli* were multidrug resistant (MDR). Similarly, among gram negative nonfermenters, 73.3% of* Acinetobacter* spp. and 41% of* Pseudomonas* spp. were MDR. About 42.8% of* Klebsiella* spp., 30% of* E. coli*, 16.7% of* Acinetobacter*, and 12.8% of* Pseudomonas* spp. were ESBL producers. In addition to this, 10% of* E. coli*, 28.5% of* Klebsiella* spp., and 6.6% of* Acinetobacter* spp. were MBL producers. The proportion of MDR isolates was significantly varied in the pediatric (13.3%) and adult (29.0%) patients (*p* < 0.005, CI-95%) (Table 7).

### 3.7. Trends of Resistance Determinants in Gram Positive Isolates

Drug resistance was also common among gram positive isolates. About 60% of* S. aureus* and 44.4% of* Enterococcus* spp. were multidrug resistant. Methicillin resistance and inducible clindamycin resistance (iMLS_B_) were observed in 31.4% and 10% of* Staphylococcus aureus* isolates, respectively. However, incidence of multidrug resistant gram positive bacteria causing BSI among pediatric and adult patients was not significantly different (Table 8).

## 4. Discussion

Bloodstream infections remained a challenge for the infectious disease physicians due to the changing bacterial etiology and emergence of antimicrobial resistance. Early detection of causative organism and determination of its antimicrobial susceptibility profile are necessary to help clinicians decide appropriate empirical therapy, which ultimately decreases the emergence of resistance [16]. Our study evaluates the incidences of bloodstream infections, bacterial etiology, and antimicrobial susceptibilities among the pediatric and adult group of patients and confirms that there is significant variation among these parameters within these groups of patient. Our data emphasizes the dominance of antibiotic resistant bacteria causing BSIs in both groups of patients and this trend is ever increasing.

In this study, overall incidence of bloodstream infection based on significant bacterial growth in the blood cultures obtained from suspected patients was 7.48%. Comparatively, similar rates of BSI have been reported from the studies of Sharma et al. (6.9%), Pandey et al. (12.6%), and Shrestha et al. (13.3%) from nearby hospitals in Kathmandu, Nepal [17–19]. Similar rates of BSI were also documented in other studies of this region, particularly by Easow et al. (10.2%) from Pokhara, Singh et al. (10.16%) and Gupta and Kashyap (16.5%) from India, and Qureshi and Aziz (16.6%) from Pakistan [10, 11, 20, 21]. Our findings of BSI are lower when compared to the reported rates by Amatya et al. (23.1%), Alam et al. (20.9%), Arora and Devi (20.02%), and Fayyaz et al. (20.0%), respectively [22–25]. The variation in the BSI rates among these studies may be attributable to sampling volume of blood culture, culture system, and medium formulation as well as type of patients enrolled in the study. Furthermore, lower rates of BSI may be ascribed to the injudicious use of antibiotics not only by clinicians before referring to the tertiary care center but also by patients themselves.

Bloodstream infections varied significantly within age groups, where the highest prevalence was recorded among patients at the lower extreme of ages: infants (12.9%), children (6.9%), adults (6.72%), and elderly (5.5%). Pradhan et al. also reported the similar rates of BSI in pediatric population in their study [26]. In this study, more pediatric patients (9.3%) were found with BSI as compared to adult patients (6.6%) and this difference was statistically significant (*p* < 0.05). Unfortunately, we could not evaluate these trends with other previous studies due to unavailability of published literature from Nepal comparing these trends. Furthermore, disparity between the BSI rates among various age groups could not be justified because the risk factors associated with BSI were not evaluated. However, it is generally accepted that infants are more likely to have BSI compared with patients of higher ages. Infants have poor skin integrity with developing immune system and they frequently participate in activities that predispose them to external environment making them prone to bacterial infections [5].

Gram negative bacteria (65.8%) were found to be the predominant cause of bloodstream infection in this study, but the majority of bacterial findings were* Staphylococcus aureus*. Gram positive bacteria were significantly associated with pediatric BSI, while gram negative isolates were significant in adult BSI cases (*p* < 0.05).* Staphylococcus aureus* (*n* = 41, 45.6%) was the most common among pediatric patients while* Salmonella enterica* (*n* = 40, 28.3%) was the most common among adult patients (*p* < 0.05). This finding is consistent with the previous findings of Pradhan et al. [26], Amatya et al. [22], Sharma et al. [17], and Easow et al. [20]. However, Shrestha et al. [19] from Nepal, Arora and Devi from India [24], and Moyo et al. from Tanzania [27] reported the gram positive bacterial dominance in blood stream infections. Over the time, etiology of BSI is continuously changing and BSI in our study was largely due to gram negative bacteria, but significant contribution from gram positive isolates, mostly* S. aureus,* was remarkable. We believe that the variation might be due to difference in geographical location, nature of patient population, endemicity of the etiological agents, and seasonal variation.

*Salmonella enterica* associated bloodstream infections were the most common gram negative bacteremic cases in our study, which suggests enteric fever is still a major problem in our region [17, 18, 28]. This trend is also consistent with previous studies from Nepal [18, 20], India [10], Pakistan [21], and Africa [5]. In our study, all the cases of enteric fever were community acquired. We isolated* Salmonella* spp. from 21.2% of the patients with BSIs, while a previous study from western Nepal reported* Salmonella* associated blood culture positivity in 51.6% of suspected cases [17]. Notably, more (55.1%)* S*. Paratyphi were responsible for enteric fever cases than *S*. Typhi in this study which may have future implication on the vaccine strategies, as the polyvalent vaccines against typhoid and paratyphoid fever are unavailable in this region [29, 30].* Escherichia coli*,* Klebsiella* spp., and* Pseudomonas* and* Acinetobacter* spp. were other common gram negative isolates in this study which is similar to the findings of previous studies from this region [18, 19, 26]. A high prevalence of nonfermenters in this study, particularly* Pseudomonas* spp. (16.8%) and* Acinetobacter* spp. (12.9%), highlights the significant concern of hospital acquired BSI in our region.

The antibiotic susceptibility spectrum of bacterial pathogens significantly varies according to the patient population, their age, and the strains. In this study,* Salmonella enterica* isolates were noted to be susceptible to most of the routinely used antibiotics. However, increased resistance of* Salmonella enterica* isolates towards ampicillin (14.2%) and fluoroquinolones (pediatric 77.7% and adults 87.5%) has compromised the therapeutic choices. Nalidixic acid resistance (NAR) was documented in 85.7% of the* Salmonella* isolates which might be the principal cause of fluoroquinolones resistance in this study [19]. In developing countries, including Nepal, ampicillin and fluoroquinolones are still the drugs of choice for treatment of enteric fever. The occurrence of drug resistance among these isolates is of great concern. Although there were no MDR* Salmonella* isolates in our study, previous reports of Pokharel et al. (5%) and Khanal et al. (26%) have documented the existence of MDR* Salmonella* in Nepal [9, 31].

Broad spectrum antibiotics, notably cephalosporins and quinolones, are the mainstay for therapy for undifferentiated febrile illness in Nepalese hospitals citing their low toxicity and higher effectiveness [18, 19]. Irrational and increased use of these antibiotics resulting in upsurge of multiple drug resistance in microorganisms is an emerging problem [32]. Most of the isolates (42.8%) found in our study were multidrug resistant (MDR) including gram positive bacteria (58.2%) and gram negative bacteria (34.8%), respectively, and this trend was significantly high among adult patients. Similar rates of multiple drug resistant bacteria were reported from an Indian study [10]. Among* Staphylococcus aureus*, increased resistance was observed in ampicillin (80% and 100%), cotrimoxazole (26.6% and 84.0%), erythromycin (51.1% and 84.0%), and ciprofloxacin (25.0% and 96.7%) among pediatric and adult patients, respectively. Methicillin resistant* Staphylococcus aureus* (MRSA) was found in significant frequency (31.4%), almost similar to the findings of previous studies [10, 33, 34]. Isolation of MRSA strains, particularly from pediatric cases of BSI, is a serious issue as therapeutic choices become very limited in these cases [33]. Further,* Enterococcus* spp. in BSI cases in this study were comparatively few but a higher number (44.4%) of them were MDR which is similar to an Indian study [10].

Among gram negative isolates, overall antibiotic susceptibility pattern suggests a high proportion of MDR organism in our hospital. In this study, we found 34.5% of gram negative isolates to be MDR. Higher proportion (48.2%) of MDR organisms was responsible for BSI cases in adults as compared to pediatric patients (30.0%) and this trend was statistically significant (*p* < 0.05). Higher resistance was observed among the ampicillin (up to 100%), fluoroquinolones (up to 83%), and broader spectrum cephalosporins (up to 66.7%). The fact that cephalosporins are one of the most commonly used antibiotics for inpatients as well as for outpatients could be the reason for such high level of resistance being observed in the developing countries. Similar patterns of susceptibilities were found in other studies from South Asian region [10, 25]. Gram negative isolates resistant to broad spectrum cephalosporins and carbapenems were also documented in this study. Beta-lactam antibiotic resistance among gram negative bacteria to antibiotics is often associated with the production of hydrolytic enzymes particularly extended spectrum *β*-lactamases (ESBLs), class C cephalosporinase (AmpC), and carbapenemases (including Metallo-beta-lactamases) [35]. The high level of cephalosporin resistance observed in our study has been supported by higher rates of beta-lactamase producing bacterial strains. Among total 152 gram negative isolates in this study, 12.5% were producing ESBL and 3.9% producing MBL. The higher rates of ESBL and MBL documented in* Klebsiella* spp. (42.8% and 28.5%) were similar to the findings of a previous study [36]. Furthermore, previous reports of beta-lactamase producing organisms associated with various bacterial infections from Nepal have also highlighted the rising scenario of dissemination of these superbugs in hospital as well as in the community [37, 38]. The greatest threat with MDR, ESBL, and carbapenemase producing gram negative bacteria is that bacterial infections including bacteremia are becoming untreatable due to the limited therapeutic options of the antibiotics available, resulting into increased mortality and health care resources [39]. Therefore, we believe this report would be helpful in encouraging the physicians to discontinue the irrational use of antibiotics and controlling the occurrence and the spread of resistance.

## 5. Limitations

The study has some limitations. We could not rule out the prior use of antimicrobial drugs among patients; instead the study relied on information provided by the patients or their guardians. In addition, the conventional blood culture system and single blood culture specimen could have produced in less sensitivity. Risk factors and associated clinical outcomes were also not evaluated. Molecular characterization of the resistant phenotypes and their epidemiology would be more significant in public health perspective.

## 6. Conclusion

This study provides some insight into the local trends and bacterial etiology of bloodstream infections among pediatric and adult patients. Gram negative bacteria are the major contributors of BSI in our patients. A higher rate of antimicrobial resistance among gram negative and gram positive organisms is an alarming issue. Exact contributing factors for the bloodstream infections (BSI) within these groups need to be further elucidated. Rational use of antibiotics, formulation of antibiotic policy, and prompt therapy of bloodstream infections for the effective management and prevention of drug resistance are urgently needed in our setting.

## Figures and Tables

**Table 1:** Trends of bloodstream infection among various group of patients (*n* = 3,088).

Patients	Age group	Number of patients (%)	Number of patients with BSI (%)	*p*
Pediatric	Infants (<1 year)	386 (12.5%)	50 (12.9%)	*0.008*
	Children (1–14 years)	574 (18.6%)	40 (6.9%)	
Adults	Adults (15–59 years)	1949 (63.2%)	131 (6.7%)	
	Elderly (60 years or above)	179 (5.7%)	10 (5.5%)	
*Total*		*3,088*	*231 (7.48%)*	

**Table 2:** Trends of bacterial isolates associated with bloodstream infections among pediatric and adult patients (*n* = 231).

Bacterial isolates	Number (%)	Pediatric patients (*n* = 90)		Adult patients (*n* = 141)		*p*
		Outpatients (*n* = 28)	Inpatients (*n* = 62)	Outpatients (*n* = 50)	Inpatients (*n* = 91)	
Gram positive isolates	79 (34.2)	14 (50.0)	35 (56.4)	8 (16.0)	22 (24.1)	
* Staphylococcus aureus*	70 (30.3)	13 (46.5)	32 (51.6)	8 (16.0)	17 (18.6)	**0.000**
* Enterococcus* spp.	9 (3.9)	1 (3.5)	3 (4.8)	0 (0.0)	5 (5.4)	0.492
Gram negative isolates	152 (65.8)	14 (50.0)	27 (43.6)	42 (84.0)	69 (75.9)	
* Salmonella enterica*	49 (21.2)	9 (32.1)	0 (0.0)	37 (74.0)	3 (3.2)	**0.001**
* Escherichia coli*	20 (8.6)	4 (14.2)	4 (6.4)	5 (10.0)	7 (7.6)	0.550
* Klebsiella pneumoniae*	7 (3.0)	0 (0.0)	3 (4.8)	0 (0.0)	4 (4.3)	0.559
* Pseudomonas aeruginosa*	39 (16.8)	0 (0.0)	8 (12.9)	0 (0.0)	31 (34.0)	**0.007**
* Acinetobacter* spp.	30 (12.9)	0 (0.0)	9 (14.5)	0 (0.0)	21 (23.0)	0.191
* Citrobacter* spp.	4 (1.7)	1 (3.5)	1 (1.6)	0 (0.0)	2 (2.1)	0.507
* Enterobacter* spp.	3 (1.3)	0 (0.0)	2 (3.3)	0 (0.0)	1 (1.0)	0.336

**Table 3:** Trends of antimicrobial susceptibilities of gram negative isolates (excluding *Salmonella*) among pediatric and adult patients (*n* = 96).

		AMP	COT	CIP	LEV	CFM	CAZ	GEN	AK	IPM	MRP	PIT	C	PB
*Escherichia coli *(*n* = 20)	Pediatric (*n* = 8)	4 (50.0)	2 (25.0)	0 (0.0)	0 (0.0)	2 (25.0)	2 (25.0)	4 (50.0)	0 (0.0)	0 (0.0)	0 (0.0)	2 (25.0)	0 (0.0)	—
	Adults (*n* = 12)	8 (66.7)	4 (33.3)	10 (83.3)	10 (83.3)	8 (66.7)	8 (66.7)	4 (33.3)	4 (33.3)	2 (16.7)	2 (16.7)	2 (16.7)	4 (33.3)	—

*Klebsiella *spp. (*n* = 7)	Pediatric (*n* = 3)	3 (100)	2 (66.7)	2 (66.7)	1 (33.3)	1 (33.3)	0 (0.0)	1 (33.3)	0 (0.0)	0 (0.0)	0 (0.0)	1 (33.3)	0 (0.0)	—
	Adults (*n* = 4)	4 (100)	3 (75)	3 (75.0)	3 (75.0)	3 (75.0	3 (75.0	3 (75.0	3 (75.0)	2 (50.0)	2 (50.0)	2 (50.0)	2 (50.0)	—

*Pseudomonas *spp. (*n* = 39)	Pediatric (*n* = 8)	—	—	2 (25.0)	1 (12.5)	—	1 (12.5)	2 (25.0)	1 (12.5)	0 (0.0)	0 (0.0)	1 (12.5)	0 (0.0)	0 (0.0)
	Adults (*n* = 31)	—	—	30 (96.7)	27 (87.0)	—	6 (19.3)	21 (67.7)	18 (58.0)	7 (22.5)	7 (22.5)	9 (29.0)	4 (12.9)	0 (0.0)

*Acinetobacter *spp. (*n* = 30)	Pediatric (*n* = 9)	—	5 (55.5)	4 (44.4)	2 (22.2)	4 (44.4)	4 (44.4)	5 (55.5)	3 (33.3)	1 (11.1)	1 (11.1)	1 (11.1)	3 (33.3)	—
	Adults (*n* = 21)	—	11 (52.3)	10 (47.2)	8 (38.1)	14 (66.7)	14 (66.7)	8 (38.1)	8 (38.1)	5 (23.8)	5 (23.8)	5 (23.8)	8 (38.1)	—

AMP = ampicillin, COT = cotrimoxazole (trimethoprim + sulfamethoxazole), CIP = ciprofloxacin, LEV = levofloxacin, CFM = cefixime, CAZ = ceftazidime, GEN = gentamycin, AK = amikacin, IPM = imipenem, MRP = meropenem, PIT = piperacillin + tazobactam, C = chloramphenicol, PB = polymyxin.

**Table 4:** Trends of antimicrobial susceptibilities of *Salmonella enterica* clinical isolates (*n* = 49).

Antimicrobial agents	*Salmonella enterica *serotype Typhi (*n* = 22)		*Salmonella enterica *serotype Paratyphi (*n* = 27)	
	Resistance (%)		Resistance (%)	
	Pediatric (*n* = 5)	Adults (*n* = 17)	Pediatric (*n* = 4)	Adults (*n* = 23)
Ampicillin	0 (0.0)	2 (11.7)	1 (25.0)	4 (17.3)
Cotrimoxazole	0 (0.0)	0 (0.0)	0 (0.0)	0 (0.0)
Ciprofloxacin	4 (80.0)	12 (70.5)	3 (75.0)	23 (100.0)
Cefixime	0 (0.0)	0 (0.0)	0 (0.0)	0 (0.0)
Ceftriaxone	0 (0.0)	0 (0.0)	0 (0.0)	0 (0.0)
Chloramphenicol	0 (0.0)	0 (0.0)	0 (0.0)	0 (0.0)
Azithromycin	0 (0.0)	0 (0.0)	0 (0.0)	0 (0.0)

**Table 5:** Trends of antimicrobial susceptibilities of gram positive isolates (*n* = 79).

Antimicrobial agents	*Staphylococcus aureus *(*n* = 70)		*Enterococcus *(*n* = 9)	
	% resistance		% resistance	
	Pediatric (*n* = 45)	Adults (*n* = 25)	Pediatric (*n* = 4)	Adults (*n* = 5)
Ampicillin	36 (80.0)	25 (100)	1 (25.0)	4 (80.0)
Cotrimoxazole	12 (26.6)	21 (84.0)	—	—
Ciprofloxacin	11 (24.4)	22 (88.0)	2 (50.0)	2 (40.0)
Cephalexin	17 (37.7)	11 (44.0)	—	—
Cloxacillin	17 (37.7)	9 (36.0)	—	—
Erythromycin	23 (51.1)	21 (84.0)	—	—
Levofloxacin	9 (20.9)	17 (68.0)	1 (25.0)	3 (60.0)
Amikacin	4 (8.9)	11 (44.0)	2 (50.0)	2 (40.0)
Clindamycin	23 (51.14)	20 (80.0)	—	—
Piperacillin-tazobactam	8 (17.7)	6 (24.0)	1 (25.0)	3 (60.0)
Imipenem	17 (37.7)	9 (36.0)	—	—
Teicoplanin	0 (0.0)	0 (0.0)	0 (0.0)	0 (0.0)

**Table 6:** Trends of multidrug resistance of bacterial isolates among pediatric and adult patients (*n* = 231).

Bacterial isolates	Total number (%)	Pediatric patients (*n* = 90)	Adult patients (*n* = 141)	*p* *value*
Total MDR isolates (GNB)	53 (34.8)	12 (29.2)	41 (36.9)	0.346
Total ESBL isolates (GNB)	19 (12.5)	8 (19.5)	11 (7.8)	0.136
Total MBL isolates (GNB)	6 (3.9)	1 (2.4)	5 (4.5)	0.433
Total MDR isolates (GPC)	43 (54.4)	17 (34.6)	26 (86.6)	**0.000**

*Total MDR isolates *	*96 (41.5)*	*29 (32.2)*	*67 (47.5)*	*0.020*

GNB = gram negative bacilli, GPC = gram positive cocci.

**Table 7:** Trends of antimicrobial resistance mechanisms in gram negative isolates among pediatric and adult patients (*n* = 231).

Bacterial isolates	Total number (%)	Pediatric patients (*n* = 90)	Adult patients (*n* = 141)	*p value*
*Salmonella enterica n* = 49				
MDR	0 (0.0)	0 (0.0)	0 (0.0)	—
NARS	42 (85.7)	7 (77.7)	35 (87.5)	0.381
*Escherichia coli n* = 20				
MDR	10 (50.0)	3 (37.5)	7 (58.3)	0.325
ESBL	6 (30.0)	2 (25.0)	4 (33.3)	0.545
MBL	2 (10.0)	0 (0.0)	2 (16.7)	0.347
*Klebsiella* spp. *n* = 7				
MDR	5 (71.4)	1 (33.3)	4 (100)	0.143
ESBL	3 (42.8)	1 (33.3)	2 (50.0)	0.629
MBL	2 (28.5)	0 (0.0)	2 (50.0)	0.286
*Pseudomonas* spp. *n* = 39				
MDR	16 (41.0)	2 (25.0)	14 (45.1)	0.269
ESBL	5 (12.8)	2 (25.0)	3 (9.6)	0.268
MBL	0 (0.0)	0 (0.0)	0 (0.0)	—
*Acinetobacter* spp. *n* = 30				
MDR	22 (73.3)	6 (66.6)	16 (76.1)	0.453
ESBL	5 (16.7)	3 (33.3)	2 (9.5)	0.143
MBL	2 (6.6)	1 (11.1)	1 (4.7)	0.517

GNB: gram negative bacteria.

**Table 8:** Trends of antimicrobial resistance mechanisms in gram positive isolates among pediatric and adult patients (*n* = 79).

Bacterial isolates	Number (%)	Pediatric patients (*n* = 90)	Adult patients (*n* = 141)	*p value*
*Staphylococcus aureus n* = 70				
MDR	42 (60.0)	16 (35.5)	23 (92.0)	**0.000**
MRSA	22 (31.4)	12 (17.1)	10 (14.3)	0.188
iMLS_B_	7 (10.0)	3 (4.2)	4 (5.8)	0.201
*Enterococcus *spp. *n* = 9				
MDR	4 (44.4)	1 (11.1)	3 (33.3)	0.357

## Abbreviations

ASM: American Society for Microbiology
ATCC: American type culture collection
BHI: Brain heart infusion
BSI: Bloodstream infection
CDT: Combined disk test
CLSI: Clinical and Laboratory Standards Institute
CAZ: Ceftazidime
CTX: Cefotaxime
iMLS_B_: Inducible macrolide-lincosamide-streptogramin-B
ESBL: Extended spectrum *β*-lactamase
MBL: Metallo-*β*-lactamase
MRSA: Methicillin resistant* Staphylococcus aureus*
MDR: Multidrug resistant
ZOI: Zone of inhibition.

## Additional Points

*Availability of Data and Materials*. The primary raw data will be made available to the interested researchers by the corresponding author of this article if requested.

## References

[1] Viscoli C. (2016). Bloodstream Infections: the peak of the iceberg. *Virulence*.

[2] Aung A. K., Skinner M. J., Lee F. J., Cheng A. C. (2012). Changing epidemiology of bloodstream infection pathogens over time in adult non-specialty patients at an Australian tertiary hospital. *Communicable Diseases Intelligence Quarterly Report*.

[3] Laupland K. B. (2013). Incidence of bloodstream infection: a review of population-based studies. *Clinical Microbiology and Infection*.

[4] Munford R. S., Suffredini A. F., Mandell G. L., Bennett J. E., Dolin R. (2010). Sepsis, severe sepsis, and septic shock. *Mandell, Douglas and Bennett's Principle and Practice of Infectious Diseases. Volume 1*.

[5] Obeng-Nkrumah N., Labi A.-K., Addison N. O., Labi J. E. M., Awuah-Mensah G. (2016). Trends in paediatric and adult bloodstream infections at a Ghanaian referral hospital: a retrospective study. *Annals of Clinical Microbiology and Antimicrobials*.

[6] Trevion S., Ross D., Mahon C. R., Lehman D. C., Manuselis G. (2007). Bacteremia and septicemia. *Textbook of Diagnostic Microbiology*.

[7] Wilson J., Elgohari S., Livermore D. M. (2011). Trends among pathogens reported as causing bacteraemia in England, 2004–2008. *Clinical Microbiology and Infection*.

[8] Dramowski A., Cotton M. F., Rabie H., Whitelaw A. (2015). Trends in paediatric bloodstream infections at a South African referral hospital. *BMC Pediatrics*.

[9] Pokharel B. M., Koirala J., Dahal R. K., Mishra S. K., Khadga P. K., Tuladhar N. R. (2006). Multidrug-resistant and extended-spectrum beta-lactamase (ESBL)-producing Salmonella enterica (serotypes Typhi and Paratyphi A) from blood isolates in Nepal: surveillance of resistance and a search for newer alternatives. *International Journal of Infectious Diseases*.

[10] Gupta S., Kashyap B. (2016). Bacteriological profile and antibiogram of blood culture isolates from a tertiary care hospital of North India. *Tropical Journal of Medical Research*.

[11] Singh A. K., Venkatesh V., Singh R. P., Singh M. (2014). Bacterial and antimicrobial resistance profile of bloodstream infections: a hospital-based study. *CHRISMED Journal of Health and Research*.

[12] Isenberg H. D. (2004). *Clinical Microbiology Procedure Handbook*.

[13] CLSI (2015). Performance standards for antimicrobial susceptibility testing; twenty-fifth informational supplement. *CLSI Document*.

[14] Magiorakos A.-P., Srinivasan A., Carey R. B. (2012). Multidrug-resistant, extensively drug-resistant and pandrug-resistant bacteria: an international expert proposal for interim standard definitions for acquired resistance. *Clinical Microbiology and Infection*.

[15] Yong D., Lee K., Yum J. H., Shin H. B., Rossolini G. M., Chong Y. (2002). Imipenem-EDTA disk method for differentiation of metallo-*β*-lactamase-producing clinical isolates of *Pseudomonas* spp. and *Acinetobacter* spp.. *Journal of Clinical Microbiology*.

[16] Penno E. C., Baird S. J., Crump J. A. (2015). Cost-effectiveness of surveillance for bloodstream infections for sepsis management in low-resource settings. *American Journal of Tropical Medicine and Hygiene*.

[17] Sharma N. P., Peacock S. J., Phumratanaprapin W., Day N., White N., Pukrittayakamee S. (2006). A hospital-based study of bloodstream infections in febrile patients in Dhulikhel Hospital Kathmandu University Teaching Hospital, Nepal. *Southeast Asian Journal of Tropical Medicine and Public Health*.

[18] Pandey S., Raza S., Bhatta C. P. (2013). The aetiology of the bloodstream infections in the patients who presented to a tertiary care teaching hospital in Kathmandu, Nepal. *Journal of Clinical and Diagnostic Research*.

[19] Shrestha S., Amatya R., Shrestha R. K., Shrestha R. (2014). Frequency of blood culture isolates and their antibiogram in a teaching hospital. *Journal of the Nepal Medical Association*.

[20] Easow J. M., Joseph N. M., Dhungel B. A., Chapagain B., Shivananda P. G. (2010). Blood stream infections among febrile patients attending a teaching hospital in Western Region of Nepal. *Australasian Medical Journal*.

[21] Qureshi M., Aziz F. (2011). Prevalence of Microbial isolates in Blood Cultures and their Antimicrobial Susceptibility Profiles. *Biomedica*.

[22] Amatya N. M., Shrestha B., Lekhak B. (2007). Etiological agents of bacteraemia and antibiotic susceptibility pattern in Kathmandu Model Hospital. *Journal of the Nepal Medical Association*.

[23] Alam M. S., Pillai P. K., Kapur P., Pillai K. K. (2011). Resistant patterns of bacteria isolated from bloodstream infections at a university hospital in Delhi. *Journal of Pharmacy and Bioallied Sciences*.

[24] Arora U., Devi P. (2007). Bacterial profile of blood stream infections and antibiotic resistance pattern of isolates. *JK Science*.

[25] Fayyaz M., Mirza I. A., Ikram A., Hussain A., Ghafoor T., Shujat U. (2013). Pathogens causing blood stream infections and their drug susceptibility profile in immunocompromised patients. *Journal of the College of Physicians and Surgeons Pakistan*.

[26] Pradhan R., Shrestha U., Gautam S. C. (2012). Bloodstream infection among children presenting to a general hospital outpatient clinic in urban Nepal. *PLoS ONE*.

[27] Moyo S., Aboud S., Kasubi M., Maselle S. Y. (2010). Bacteria isolated from bloodstream infections at a tertiary hospital in Dar es Salaam, Tanzania—antimicrobial resistance of isolates. *South African Medical Journal*.

[28] Prajapati B., Rai G. K., Rai S. K. (2008). Prevalence of Salmonella typhi and paratyphi infection in children: a hospital based study. *Nepal Medical College Journal*.

[29] Bajracharya D., Khan M. I., Pach A. (2014). 25 Years after Vi typhoid vaccine efficacy study, typhoid affects significant number of population in Nepal. *PLoS ONE*.

[30] Karkey A., Thompson C. N., Tran Vu Thieu N. (2013). Differential epidemiology of Salmonella Typhi and Paratyphi A in Kathmandu, Nepal: a matched case control investigation in a highly endemic enteric fever setting. *PLoS Neglected Tropical Diseases*.

[31] Khanal B., Sharma S. K., Bhattacharya S. K., Bhattarai N. R., Deb M., Kanungo R. (2007). Antimicrobial susceptibility patterns of *Salmonella enterica* serotype typhi in eastern Nepal. *Journal of Health, Population and Nutrition*.

[32] Mehta M., Dutta P., Gupta V. (2005). Antimicrobial susceptibility pattern of blood isolates from a teaching hospital in North India. *Japanese Journal of Infectious Diseases*.

[33] Mishra S. K., Rijal B. P., Pokhrel B. M. (2013). Emerging threat of multidrug resistant bugs—acinetobacter calcoaceticus baumannii complex and Methicillin resistant Staphylococcus aureus. *BMC Research Notes*.

[34] Vanitha R. N., Kannan G., Venkata N. M., Vishwakanth D., Nagesh V. R., Yogitha M. (2012). A retrospective study on blood stream infections and antibiotic susceptibility patterns in a tertiary care teaching hospital. *International Journal of Pharmacy and Pharmaceutical Sciences*.

[35] Bush K. (2010). Bench-to-bedside review: the role of *β*-lactamases in antibiotic-resistant Gram-negative infections. *Critical Care*.

[36] Al-Mulla N. A., Taj-Aldeen S. J., El Shafie S., Janahi M., Al-Nasser A. A., Chandra P. (2014). Bacterial bloodstream infections and antimicrobial susceptibility pattern in pediatric hematology/oncology patients after anticancer chemotherapy. *Infection and Drug Resistance*.

[37] Mishra S. K., Awal B. K., Kattel H. P. (2014). Drug resistant bacteria are growing menace in a University Hospital in Nepal. *American Journal of Epidemiology and Infectious Disease*.

[38] Ansari S., Nepal H. P., Gautam R. (2015). Community acquired multi-drug resistant clinical isolates of Escherichia coli in a tertiary care center of Nepal. *Antimicrobial Resistance and Infection Control*.

[39] WHO (2014). *Antimicrobial Resistance: Global Report on Surveillance*.

